# Nucleus, Mitochondrion, or Reticulum? STAT3 à La Carte

**DOI:** 10.3390/ijms19092820

**Published:** 2018-09-18

**Authors:** Lidia Avalle, Valeria Poli

**Affiliations:** Department of Molecular Biotechnology and Health Sciences, Molecular Biotechnology Center, University of Turin, Via Nizza 52, 10126 Turin, Italy

**Keywords:** STAT3, post-translational modifications, endoplasmic reticulum, mitochondrial associated membranes (MAMs), nucleus, apoptosis, cancer

## Abstract

The transcription factor signal transducer and activator of transcription (STAT)3 mediates the functions of cytokines, growth factors, and oncogenes under both physiological and pathological conditions. Uncontrolled/constitutive STAT3 activity is often detected in tumors of different types, where its role is mostly that of an oncogene, contributing in multiple ways to tumor transformation, growth, and progression. For this reason, many laboratories and pharmaceutical companies are making efforts to develop specific inhibitors. However, STAT3 has also been shown to act as a tumor suppressor in a number of cases, suggesting that its activity is strongly context-specific. Here, we discuss the bases that can explain the multiple roles of this factor in both physiological and pathological contexts. In particular, we focus on the following four features: (i) the distinct properties of the STAT3α and β isoforms; (ii) the multiple post-translational modifications (phosphorylation on tyrosine or serine, acetylation and methylation on different residues, and oxidation and glutathionylation) that can affect its activities downstream of multiple different signals; (iii) the non-canonical functions in the mitochondria, contributing to the maintenance of energy homeostasis under stress conditions; and (iv) the recently discovered functions in the endoplasmic reticulum, where STAT3 contributes to the regulation of calcium homeostasis, energy production, and apoptosis.

## 1. Introduction

Signal transducer and activator of transcription (STAT) factors are activated by tyrosine phosphorylation, mainly by receptor-associated Janus kinases (JAKs) downstream of cytokine and growth factor receptors [1,2]. Tyrosine-phosphorylated (YP)-STATs active dimers, formed by reciprocal SH2 domain-phosphotyrosine interaction, concentrated in the nucleus to regulate the expression of target genes [3]. The ubiquitously expressed STAT3 family member is canonically activated by YP downstream of a wide variety of cytokines, including those of the interleukin-6 (IL-6) family, leptin, IL-12, IL-17, and IL-10, as well as interferons; growth factors, such as granulocyte colony-stimulating factor (G-CSF), epidermal growth factor (EGF), platelet-derived growth factor (PDGF), and oncogenes like Src, Abl, Sis, Fps, Ros, Met, and ErbB2 [4,5,6]; G protein coupled receptors like the sphingosine-1-phosphate receptor (S1PR)1; and Toll-like receptors (for recent reviews, see [6,7]). Accordingly, STAT3 is known to play a multitude of distinct and sometimes even contradictory functions in different cell types, both under physiological and pathological conditions. It is thus not surprising that STAT3 gene inactivation, which is unlikely for that of all other STAT genes, is early embryonic lethal [8]. Indeed, STAT3 plays an important role in cell growth and survival. Among its numerous functions are the induction of liver acute-phase response genes and of liver regeneration [9,10], the stimulation of B lymphocytes differentiation [11], the regulation of the activity of many hematopoietic cells subsets [12,13,14,15,16,17], the induction of lysosome-mediated apoptosis during mammary gland involution [18], the protection from apoptosis and heart infarction [19], and the maintenance of pluripotency in embryonic stem cells [20]. Most of these functions were exhaustively described in recent reviews, including some published as part of this Special Issue [21,22,23,24,25,26,27].

The activation/inactivation of STAT proteins is a crucial step for their biological activities, and the disruption of this control, leading to defective or constitutive STATs activation, usually correlates with the development of pathological conditions. Accordingly, STAT3 activation must be tightly controlled by negative regulators, which mainly belong to three groups, namely: phosphatases, suppressor of cytokine signaling (SOCS) proteins, and the protein inhibitor of activated STAT (PIAS) proteins [28,29,30]. Phosphatases, like SHP-1, SHP-2, and PTP1B, directly counteract JAK-mediated phosphorylation, whereas nuclear phosphatases, such as T-cell PTP, play a role in signal termination [28]. SOCS genes, and in particular SOCS3, are direct STAT3 target genes and act as negative feedback regulators by triggering the proteasomal degradation of JAK kinases [31]. Finally, PIAS3 prevents the binding of STAT3 to its target DNA [32].

## 2. STAT3 as a Conditional Oncogene

In agreement with the observation that most of its numerous activators/negative regulators are frequently deregulated in cancer, STAT3 is frequently constitutively activated in many tumors both of hematopoietic and epithelial origin, and is mostly considered as an oncogene (recently reviewed in the literature [6,7,21]). Activating mutations are also detected, mostly in lymphoid tumors [33,34,35]. Importantly, both the transcriptional and non-transcriptional activities of STAT3 can promote tumorigenesis. Indeed, STAT3 transcriptional functions are required for tumor transformation by oncogenes that trigger its YP, such as, for example, Src [36]. On the other hand, STAT3 is also required for Ras-mediated transformation, independently of YP and transcriptional activity [37].

The STAT3 constitutive activity in tumors can induce proliferation and resistance to apoptosis, down-modulate immune responses and promote angiogenesis, enhance invasion and metastasis by inducing epithelial to mesenchymal transition (EMT), alter the extracellular matrix inducing matrix metalloproteinases (MMP) and promote collagen cross-linking and tissue tension, regulate energy metabolism and mitochondrial activity, and confer cancer stem cell features in a number of liquid and solid tumors [7,38,39,40,41]. Finally, STAT3 activity is also crucial to determine the pro-tumorigenic features of most tumor stromal cells [21].

However, in keeping with the complexity of its functions, recent evidence is accumulating that STAT3 can also act as a tumor suppressor under specific conditions (recently reviewed in [21]), even in tumors where it was previously shown to play pro-oncogenic functions. For example, STAT3 could inhibit glial tumor growth downstream of mutations of PTEN, but not of type III epidermal growth factor receptor (EGFRvIII) [42]; help bypass senescence in prostate cancer [43]; suppress tumor development by impairing the expression of IL-8 and thus the recruitment of myeloid cells in kirsten rat sarcoma viral oncogene homolog (K-RAS) mutant lung adenocarcinomas [44]; impair tumor progression in APC mutant colon cancer but not in inflammation-driven colorectal carcinogenesis [45,46,47,48]; and impair tumor cell proliferation in thyroid carcinomas [49]. Thus, it appears that the STAT3 functions in cancer can vary according to context, genetic background, and phase of the tumor.

## 3. STAT3 Multifaceted Functions: How, Where, and When?

Certainly, the multiple and even contrasting actions of STAT3 both under physiological and pathological conditions can be partly explained by its ability to activate different target genes in different cell types and under different conditions. This is in turn due to the cell- and context-specific accessibility of STAT3 binding sites on the genome [50], to its ability to interact with other transcription factors, and with distinct co-factors modulating or redirecting their activity (see [7,25,51], for recent reviews). Also relevant is the above discussed convergence of multiple signaling pathways on STAT3, which determines the STAT3 activation in many different contexts and downstream of multiple stimuli.

**Figure 1:**
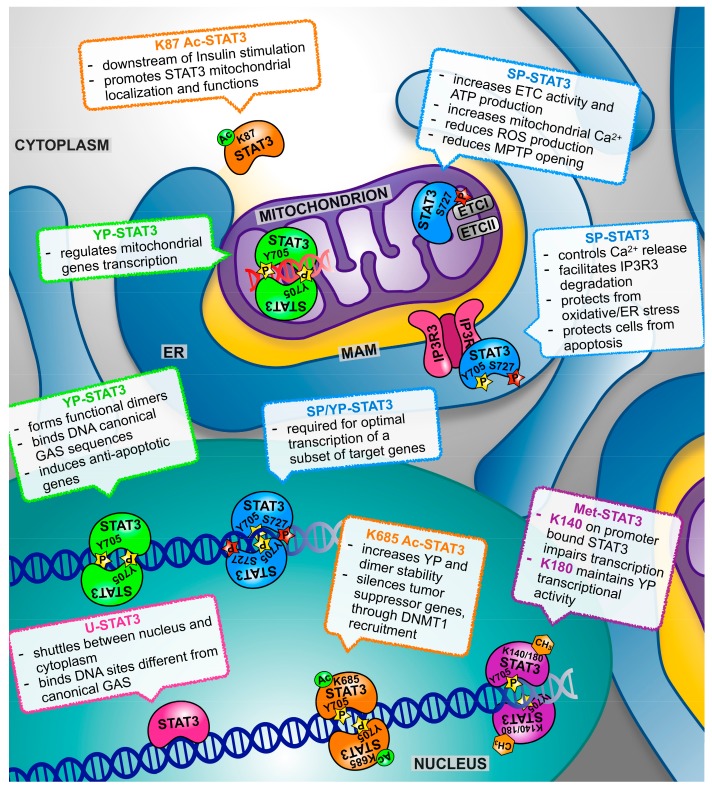
The scheme depicts the multiple roles of signal transducer and activator of transcription (STAT)3 according to its post-translational modifications and subcellular localization. See text for details and references. YP—tyrosine-phosphorylated (YP)-STATs; SP—serine phosphorylation.

On the other hand, referring to STAT3 as a single factor is somewhat misleading. Indeed, STAT3 exists in two alternatively spliced isoforms, the full length STAT3α and the truncated STAT3β, which possess both specific and overlapping transcriptional activities [52,53,54]. Additionally, STAT3 can be post-translationally modified in a number of alternative ways in addition to YP, variably affecting its activity and cellular localization (Figure 1). The main features of the α and β isoforms and of the known post-translational modifications are summarized below.

### 3.1. STAT3α and STAT3β

While STAT3α is the full-length form of STAT3, the β isoform lacks the C-terminal transcriptional activation domain (TAD) including S727, replaced by a unique seven amino acid tail [55,56]. STAT3β, which can thus get phosphorylated on Y705, and dimerize and bind to DNA, but cannot get phosphorylated on S727, has long been considered as a dominant negative form [55]. However, several works have established that STAT3β plays unique functions, activating sets of specific target genes. Notably, this isoform can alone sustain embryonal development, resulting in the post-natal death of mice specifically lacking the α isoform, in contrast to the early embryonal lethality of a complete STAT3 inactivation [52,53,54]. As recently reviewed by Aigner et al. [57], STAT3α and β apparently play the opposite functions in tumorigenesis. While STAT3α appears to be responsible for most, if not all, the pro-oncogenic activities ascribed to STAT3 [57], STAT3β can oppose these, acting as a dominant negative form [38]. In contrast however, STAT3β can behave as an oncogene, inducing T-cell leukemia development when overexpressed in transplanted bone marrow cells [58]. One of the main functions of STAT3β, however, appears to be the suppression of systemic inflammation. Indeed, STAT3β null mice are exquisitely sensitive to endotoxic shock [52,53], and develop exacerbated atherosclerosis in the absence of ApoE [59]. Consistent with this anti-inflammatory role, the mice lacking STAT3β undergo exacerbated acute inflammatory responses to irritants such as 12-*O*-Tetradecanoylphorbol-13-acetate (TPA) and dextran sodium sulfate (DSS), resulting in accelerated skin and intestinal tumorigenesis in the well-known TPA/DMBA and AOM/DSS models of inflammation-driven carcinogenesis [60]. Despite this, further tumor development is unchanged with respect to wild type mice, suggesting that STAT3β is important in maintaining tissue homeostasis, playing an anti-oncogenic role in inflammation-driven cancer mainly during the early steps of tumor formation. Thus, the balance between the two STAT3 isoforms, in turn dictated by the specific signals determining the tumor transformation and progression, and shaping the tumor microenvironment, is apparently crucial to determine the initial tumor transformation rates in inflammation-associated cancers. Unfortunately, very little information, if any, is available on the presence of other outstanding modifications that have been detected and characterized on the STAT3α isoform (see below).

### 3.2. Tyrosine Phosphorylation

As already mentioned, STAT3 canonical activity as a transcription factor is mainly due to phosphorylation on Y705, which determines the ability to form functional dimers, to concentrate in the nucleus, and to bind DNA. This occurs downstream of the many activatory signals mentioned above, and is in turn controlled by the negative regulators that lead to STAT3 dephosphorylation. However, similar to STAT1, STAT3 can regulate transcription also when not phosphorylated. Indeed, unphosphorylated (U)-STAT3 can shuttle between the nucleus and the cytoplasm [61], and has been shown to be able to interact with other factors and to directly or indirectly bind to DNA on binding sites distinct from the canonical gamma interferon activation site (GAS) sequences, thus exerting its control on a different subset of target genes, including several oncogenes [62,63,64]. Accordingly, high nuclear levels of U-STAT3 correlate with bad prognosis in glioblastoma [65]. The multiple so called “canonical” functions of nuclear and YP STAT3 have been amply reviewed elsewhere, and will not be discussed here [7,39,66].

In addition to its prevalent nuclear localization and function, YP-STAT3 has also been shown to localize to the focal adhesions in ovarian cancer cells, where it interacts with phosphorylated paxillin and focal adhesion kinase (FAK), thus regulating cell migration [67]. STAT3 can regulate cell migration also from the cytoplasm, where its non-phosphorylated form inhibits the microtubule-destabilizing protein stathmin, resulting in enhanced microtubules polymerization and cell migration [68]. Moreover, always in the cytoplasm, likely non-phosphorylated STAT3 can interact with protein kinase R (PKR), blocking its enzymatic activity and thus inhibiting autophagy [69]. This association is inhibited by fatty acids like palmitate, which leads to stimulation of the autophagic flux [69,70].

### 3.3. Serine Phosphorylation (SP)

In addition to YP, STAT3 can undergo a number of different post-translational modifications correlated with either enhanced or reduced trans-activating potential. These can however also regulate non-transcriptional functions of this factor. The first of these modifications to be detected is phosphorylation on the serine residue in position 727 (SP), located in the carboxy-terminal transcriptional activation domain and thus lacking in the STAT3β isoform. SP can be carried out by several kinases including MAP kinases, PKCε and mTOR, downstream of both classical STAT3 activating cytokines/growth factors, of the PI3 kinases and of Ras proteins [71,72]. While SP is required for the optimal transcriptional induction of a subset of target genes [73], it can also modify STAT3 activation dynamics by inhibiting the subsequent YP [71]. More recently, it became clear that SP can confer specific activities to STAT3 independently of YP, and not involving either nuclear localization or transcriptional activity. In particular, SP-STAT3 drives important functions in the mitochondria [37], as better detailed in the following sections (recently reviewed by Garama and Gough [74], and by Rincon [26]).

### 3.4. Acetylation

In response to cytokines and growth factors signaling, STAT3 can also be acetylated (Ac) on multiple lysine (K) residues by the CBP/p300 histone acetyltransferase. K685 acetylation can enhance the STAT3 transcriptional activity by increasing tyrosine phosphorylation and dimer stability [75], and K685-acetylated STAT3 was shown to silence the tumor suppressor genes by recruiting DNA methyltransferase 1 to their promoters [76]. In contrast, K87 acetylation downstream of the insulin stimulation can promote STAT3 mitochondrial translocation and functions [77]. The nicotinamide adenine dinucleotide (NAD)-dependent silent information regulator protein (SIRT)1 has been shown to be able to deacetylate STAT3 [78]. SIRT1-mediated deacetylation leads both to reduced YP/transcriptional activity and to decreased mitochondrial localization and function of SP-STAT3 [79].

### 3.5. Methylation

STAT3 methylation can occur in the nucleus on residues K140 or K180, with different effects on its activities. The histone methyltransferase SET9 is able to methylate promoter-bound STAT3 on K140, leading to impaired transcriptional activation of the target genes [80]. In contrast, the EZH2 component of the polycomb complex 2 operates K180 tri-methylation, which was shown to be required to maintain STAT3 YP and transcriptional activity in the cells from glioblastoma and prostate cancer [81].

### 3.6. Oxidation and Glutathionylation

Finally, STAT3 can become both oxidized and glutathionylated on multiple cysteine residues, impairing its transcriptional activity, either under conditions of oxidative stress or downstream of IL-6 signaling, which can raise reactive oxygen species (ROS) levels [82,83,84,85]. In contrast, mild ROS production downstream of insulin-like growth factor 1 (IGF1), EGF, and other growth factors can activate JAK kinases and enhance STAT3 YP and nuclear activity [86,87,88,89]. STAT3 oxidation can occur downstream of the cytoplasmic thiol peroxidase peroxiredoxin-2 (Prx2), one of the major H_2_O_2_ scavengers within the cell [85]. This achieves ROS detoxification while at the same time impairing IL-6-induced, STAT3-mediated transcription, suggesting that STAT3 is part of a redox relay controlling redox homeostasis, and ROS and cytokine signaling. Indeed, the expression of a redox-insensitive cysteine mutant STAT3 leads to increased STAT3 activity and cell growth rates. Therefore, this cross-talk between oxidative and non-oxidative STAT3 modifications can affect the activities of YP-STAT3 as well as cell proliferation and survival. Whether oxidation can also affect the activity of other modified STAT3 forms is presently unknown. The complex functions of STAT3 in redox homeostasis were recently reviewed [21,90].

## 4. Both Nuclear and Mitochondrial STAT3 Affect Energy Metabolism and Cellular Respiration

Tumor cells are known to rely on the so-called Warburg effect for their growth on metabolic rewiring [91] involving, among others, a switch in glucose metabolism consisting of increased aerobic glycolysis and reduced mitochondrial activity [92]. Although well known for many decades, only recently was this switch explained alongside the need for rapidly growing cells to use lactate as a building block for rapid macromolecules synthesis. Indeed, while glycolysis is energetically less favorable because of the lower moles of ATP produced per mole of glucose, provided that the glucose supply is not in shortage, glycolysis is faster than aerobic respiration. Moreover, lactate is often used as a high-energy substrate by neighboring aerobic cells [93]. We have shown that constitutively active STAT3 plays a key role in this switch, promoting aerobic glycolysis via hypoxia-inducible factor 1, α subunit (HIF1α) transcriptional induction, and down regulating mitochondrial activity via the HIF1α-independent modulation of nuclear genes encoding for electron transport complexes (ETC) [94]. This metabolic switch enhances lactate production and leads to a decreased production of ROS, protecting cells from apoptosis and senescence, and is relevant in cancer cells displaying STAT3 constitutive activity, as shown by the reduced glucose intake of tumors xenografted in mice treated with a STAT3 inhibitor [94]. Moreover, STAT3-mediated aerobic glycolysis contributes to the ability of constitutively active STAT3 to act as a hit in tumor transformation [95].

Intriguingly, as mentioned above, STAT3 was described in a number of cell types to be able to localize to mitochondria, where its SP form is able to regulate the respiratory and signaling functions of this organelle [37,96,97,98,99]. By immunoprecipitation, STAT3 was demonstrated to physically interact with distinct ETC, in primis complex 1 [37,96,100]. Accordingly, despite the lack of a classical amino-terminal mitochondrial targeting sequence, STAT3 is associated with the inner mitochondrial membrane and the mitochondrial matrix, as shown by the protease experiments [37]. It was suggested that STAT3 mitochondrial import may be mediated by Gene associated with Retinoid Interferon induced cell Mortality (GRIM) 19 [100] and/or by the heat shock protein 22, at least in cardiomyocytes [101]. Moreover, STAT3 has been shown to associate with the chaperone TOM20, leading to the hypothesis that either TOM20 or TOM70 are involved in its mitochondrial transport [74,102,103].

Mitochondrial STAT3 functions (recently reviewed by Garama and Gough [74] and by Rincon and Pereira [26]), are in line with its pro-oncogenic activities, contributing to cell survival under specific stress conditions such as the ischemic heart, or downstream of Ras-mediated transformation [37,96,104]. Specifically, mitochondrial STAT3 was shown, possibly via the interaction with ETCI and II, to preserve optimal ETC activity, increase membrane polarization and ATP production, and enhance the activity of lactate dehydrogenase. As a consequence, aerobic glycolysis is induced and ROS production decreased [96,100,104,105,106,107]. In addition to these actions, which contribute to the maintenance of energy balance under stress, protection from apoptosis could also be triggered by the inhibition of the mitochondrial permeability transition pore (MPTP) opening, which in turn may be explained by its ability to interact with cyclophilin D [102] (Figure 1). Indeed, opening of the MPTP, which is crucial for the release of pro-apoptotic peptides that initiates the intrinsic apoptotic program, is triggered by the stress conditions related to the excessive release of calcium from the endoplasmic reticulum, resulting in excessive or prolonged Ca^2+^ fluxes into the mitochondrion [108], and the STAT3 null cells were shown to require lower Ca^2+^ concentration to initiate MPTP opening [102]. Increased ETC activity is usually associated to increased ROS production, and therefore the observation of reduced mitochondrial ROS triggered by STAT3 concomitantly to increased ETC activity, confirmed in many cell types, including astrocytes and hematopoietic cells [97,98,99], appears counterintuitive. Mercedes Rincon and colleagues have proposed a brilliant answer to this conundrum, demonstrating that the activation of CD4^+^ T cell by IL-6 requires mitochondrial STAT3 and coincides with the formation of ET super complexes, which, by optimizing coupling, are known to reduce electron leakage [99]. IL-6 treatment increases the STAT3 concentration in the mitochondrion, which correlates with physical interaction with the super complexes, suggesting a role for STAT3 in their formation/function. Under these conditions, enhanced ETC activity increased the polarization of the mitochondrial membrane but, strikingly, did not trigger increase ATP production. Rather, it led to increased mitochondrial Ca^2+^ levels. In turn, these were required to ensure the prolonged stimulation of IL-4 production via the activation of the nuclear factor for activated T-cells (NFAT) and the induction of effector Th2 T-cells from naïve CD4^+^ T-cells [99]. Mitochondrial STAT3 may also contribute to the control of ROS levels via the enhanced synthesis of ROS scavengers, as it was shown that it can increase, via a still uncharacterized mechanism, the levels of the major cellular ROS scavenger glutathione, and indeed the pharmacological inhibition of GSH synthesis led to ROS accumulation, DNA damage, and cell death in STAT3-sufficient cell lines transformed by Ras [109].

SP, which was shown to occur downstream of Ras-activated MAP kinases [37,110] and of PKCε in keratinocytes upon TPA or EGF treatment [107,111], is believed to be required for most, if not all, the mitochondrial functions of STAT3. SP is however not required for STAT3 mitochondrial translocation. Rather, K87 acetylation triggered by insulin signaling was proposed to be required for STAT3 to translocate to mitochondria, where it interacts with pyruvate dehydrogenase complex E1 resulting in an increased membrane potential and ATP production [77]. On the other hand, despite not being required for the above-described functions, mitochondrial STAT3 is also amply phosphorylated on Y705 [99,107,111]. Several observations point toward a role also for YP STAT3 in this organelle. For example, BCL2 overexpression recruits YP, but not SP, and STAT3 to mitochondria in the human colon cancer cell line HCT116, where it leads to increased O_2_-production enhancing survival [112]. Additionally, an abundant signal for YP STAT3 can be observed in the mitochondrial fractions from both the activated CD4^+^ T-cells and leukemia inhibitory factor (LIF)-stimulated ES cells [99,107]. As both YP and SP are detected on mitochondrial STAT3, it would be relevant to determine whether both modifications coexist on the same molecule, or whether separate subsets of STAT3, either YP or SP, exist. YP is likely to be involved in the STAT3 transcriptional activities that have been demonstrated to occur within mitochondria. Indeed, STAT3 can bind to mitochondrial DNA in both keratinocytes and ESCs. In keratinocytes, STAT3 appears to act as a transcriptional repressor by interacting with the master mitochondrial transcription factor TFAM to down-regulate respiratory chain genes [111], leading, in contrast to all other described mitochondrial functions, to reduced ETC activity. Different is the situation in ES cells, where STAT3 acts as a transcriptional activator of transcriptional units encoding for ETC subunits, enhancing their expression levels and leading to increased complexes assembly and improved respiration, in turn a required to support ESCs proliferative activity downstream of LIF [107]. The authors conclude that this activity explains the requirement for LIF stimulation and STAT3 activity to maintain ESCs pluripotency.

Many reports suggest that several known STAT3 pro-oncogenic activities previously ascribed solely to YP involve SP instead. Originally, SP STAT3 was reported to sustain the tumor transformation of MEF cells downstream of Harvey rat sarcoma virus oncogene (H-RAS) [37]. Additionally, the SP STAT3-mediated enhancement of ETC activity, mitochondrial membrane polarization, and ATP production were also shown to be involved in K-RAS-driven myeloid malignancy and in the development of pancreatic cancer [37,103,105,110]. Moreover, SP STAT3 mediates the in vivo growth and metastatic potential of murine mammary tumor 4T1 cells by increasing complex I coupling and reducing ROS production [106]. In this vein, it has been shown that the STAT3 inhibitor phospho-valproic acid can also inhibit the mitochondrial STAT3 functions, and is able to decrease the STAT3 mitochondrial localization, correlating with the impaired growth of human pancreatic tumor xenografts [113]. Increased pituitary tumorigenesis has also been observed following expression of an FGFR form carrying the R388 single nucleotide polymorphism in pituitary cells, correlating with SP-STAT3 accumulation in mitochondria and enhanced cytochrome c oxidase activity [114].

## 5. Not only Nucleus and Mitochondrion: The Endoplasmic Reticulum Takes Centre Stage

MEF cells expressing constitutively active STAT3, in addition to a steady-state reduction of the mitochondrial membrane potential and of ETC protein levels, also featured reduced mitochondrial activation in response to ATP, assessed as a reduced flux of Ca^2+^ ions into the mitochondria [94].

**Figure 2:**
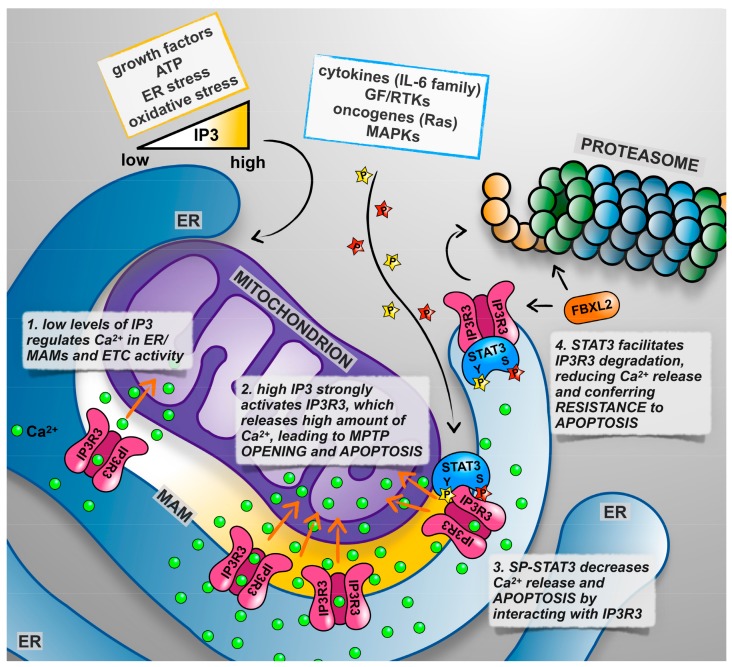
STAT3 role in the endoplasmic reticulum (ER). See text for details and references.

Ca^2+^ homeostasis and the regulation of its fluxes between different subcellular compartments are of fundamental importance for the control of many cellular functions, including life/death decisions [115]. The endoplasmic reticulum (ER) is the major intracellular Ca^2+^ storage compartment [116]. ER calcium contents are regulated by the equilibrium between the coordinated activities of Ca^2+^ pumps such as the SERCAs, which trigger ER Ca^2+^ entry, and of Ca^2+^ channels activated by the second messenger IP3 downstream of many different extracellular signals [117], the inositol 1,4,5-triphosphate receptors (IP3R). The entry of Ca^2+^ in the mitochondrial matrix regulates the activation of mitochondrial respiration, as the Ca^2+^ concentration is rate-limiting for the function of several enzymes of the Krebs cycle [118]. However, the continuous or excessive release of Ca^2+^ at the mitochondrial associated membranes (MAMs) [119], and specialized ER structures formed by the apposition of ER and mitochondrial membranes, can lead to mitochondrial Ca^2+^ overload, triggering the opening of the MPTP and the initiation of the intrinsic apoptotic program [120,121] (Figure 2). Therefore, the abundance and activity of the IP3Rs and in particular of IP3R3, which is preferentially involved in transmitting apoptotic Ca^2+^ signals to mitochondria [122], is a key factor regulating the sensitivity of cells to those apoptotic stimuli known to trigger Ca^2+^-mediated cell death, like H_2_O_2_ and menadione [123]. It is thus not surprising that the ER-mitochondria interface acts as a signaling hub for the activity of growth factors, oncogenes, and oncosuppressors [118,124]. Indeed, AKT, PTEN, PML, and BCL-2 have all been shown to regulate IP3R3 activity and be in abundance in the MAMs, leading to an altered sensitivity to apoptosis [123,125,126,127].

With this in mind, we decided to assess whether STAT3 could somehow alter Ca^2+^ release from the ER, and indeed we observed a significantly reduced ER Ca^2+^ content and release in MEF cells [128]. Importantly, the silencing of STAT3 in the STAT3-dependent MDA-MB-468 and -231 significantly increased the Ca^2+^ content and release, while not affecting at all of the MDA-MB-453 or T47D cells, which do not display activated STAT3. Increased Ca^2+^ release upon STAT3 silencing occurred, only in the STAT3-dependent cells, also upon H_2_O_2_ or menadione treatment, correlating with a significant increased sensitivity to apoptotic cell death.

A possible explanation of how STAT3 could regulate ER Ca^2+^ homeostasis came from the observation that this factor, both phosphorylated on Y705 and on S727 abundantly, localizes to the ER and MAMs, where it physically interacts with the IP3R3 Ca^2+^ channel. Although neither SP nor YP are required for either ER localization or IP3R3 interaction, it is the phosphorylation on S727 that appears to play the regulatory role, as MEF cells expressing a STAT3 mutated on S727, but not a WT or Y-F mutant, displayed excessive Ca^2+^ release and apoptotic cell death upon H_2_O_2_ or menadione treatment. Finally, we could demonstrate that ER STAT3 acts by facilitating IP3R3 proteasomal degradation, likely via the ubiquitin E3 ligase FBXL2 [127]. Indeed, IP3R3 degradation is impaired when cells are either silenced for STAT3 or only express a S-A mutant [128] (Figure 2). These observations explain why tumors often displaying STAT3 constitutive activation, such as mammary tumors belonging to the basal-like subtype, display an inverse correlation between the phospo-STAT3 and IP3R3 protein levels [128].

The well-known anti-apoptotic functions of STAT3, so far known to be based on the transcriptional activation of anti-apoptotic genes in the nucleus and on the maintenance of ETC activity in the mitochondrion, now include also its activities at the ER and MAMs, via IP3R3 degradation and modulation of Ca^2+^ fluxes.

## 6. Conclusions

STAT3 has long been considered a pleiotropic factor, due to its multiple activators and its ability to regulate the transcription of different target genes under different conditions. In the past few years, it became clear that STAT3 multifaceted functions were even underestimated, as this factor acts in an unconventional way outside from the nucleus, regulating the functions of mitochondria and the ER independently of its transcriptional activity (Figure 1). Although much has been discovered about these non-canonical functions of STAT3, our understanding of the molecular mechanisms involved is still incomplete. All of these activities contribute to the known STAT3 physiological and pathological functions, and this knowledge complicates the already difficult task of targeting STAT3 for therapeutic purposes. The question of which activity/activities should be targeted under which conditions needs to be asked. Moreover, the altered equilibrium between different STAT3 forms that could be caused by the specific inhibition of one of them needs to be taken into account.

## Abbreviations

G-CSF	Granulocyte colony-stimulating factor
EGF	Epidermal growth factor
PDGF	Platelet-derived growth factor
K-RAS	Kirsten rat sarcoma viral oncogene homolog
TPA	12-*O*-Tetradecanoylphorbol-13-acetate
DSS	Dextran Sodium Sulfate
DMBA	7,12-Dimethylbenz[a]-anthracene
AOM	Azoxymethane
GAS	Gamma interferon activation site
FAK	Focal adhesion kinase
NAD	Nicotinamide adenine dinucleotide
ROS	Reactive oxygen species
IGF1	Insulin-like growth factor 1
HIF1α	Hypoxia-inducible factor 1, α subunit
LIF	Leukemia inhibitory factor
TFAM	Mitochondrial transcription factor A
H-RAS	Harvey rat sarcoma virus oncogene
